# The Use of Bioactive Compounds in Hyperglycemia- and Amyloid Fibrils-Induced Toxicity in Type 2 Diabetes and Alzheimer’s Disease

**DOI:** 10.3390/pharmaceutics14020235

**Published:** 2022-01-20

**Authors:** Ancuta-Veronica Lupaescu, Monica Iavorschi, Mihai Covasa

**Affiliations:** 1 Department of Biomedical Sciences, College of Medicine and Biological Sciences Stefan cel Mare University of Suceava, 13 University, 720229 Suceava, Romania; ancuta.lupaescu@usm.ro (A.-V.L.); monica.iavorschi@usm.ro (M.I.); 2 Department of Basic Medical Sciences, College of Osteopathic Medicine, Western University of Health Sciences, 309E, Second Street, Pomona, CA 91777, USA

**Keywords:** amyloid peptide aggregation, insulin resistance, inflammation, neurodegenerative disease, bioactive small molecules

## Abstract

It has become increasingly apparent that defective insulin signaling may increase the risk for developing Alzheimer’s disease (AD), influence neurodegeneration through promotion of amyloid formation or by increasing inflammatory responses to intraneuronal β-amyloid. Recent work has demonstrated that hyperglycemia is linked to cognitive decline, with elevated levels of glucose causing oxidative stress in vulnerable tissues such as the brain. The ability of β-amyloid peptide to form β-sheet-rich aggregates and induce apoptosis has made amyloid fibrils a leading target for the development of novel pharmacotherapies used in managing and treatment of neuropathological conditions such as AD-related cognitive decline. Additionally, deposits of β-sheets folded amylin, a glucose homeostasis regulator, are also present in diabetic patients. Thus, therapeutic compounds capable of reducing intracellular protein aggregation in models of neurodegenerative disorders may prove useful in ameliorating type 2 diabetes mellitus symptoms. Furthermore, both diabetes and neurodegenerative conditions, such as AD, are characterized by chronic inflammatory responses accompanied by the presence of dysregulated inflammatory biomarkers. This review presents current evidence describing the role of various small bioactive molecules known to ameliorate amyloidosis and subsequent effects in prevention and development of diabetes and AD. It also highlights the potential efficacy of peptide–drug conjugates capable of targeting intracellular targets.

## 1. Introduction

**Figure 1:**
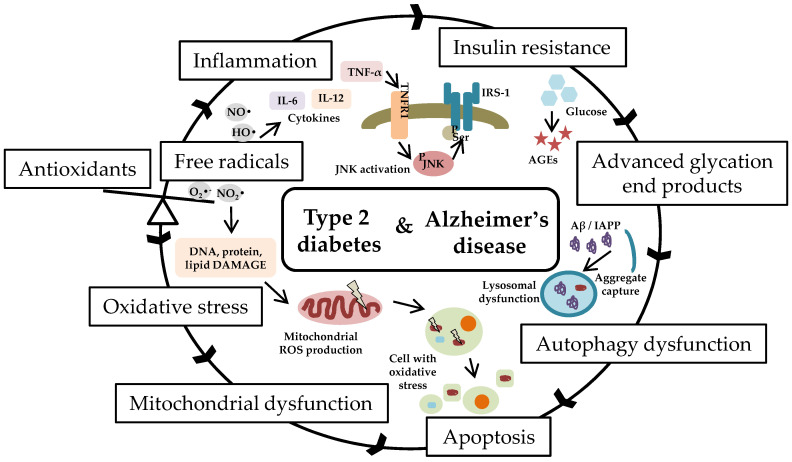
Schematic representation of the link between type 2 diabetes and Alzheimer’s disease. An imbalance between production of reactive oxygen species and the efficiency of body’s antioxidant defense system stimulates oxidative stress and inflammatory responses. Increased levels of pro-inflammatory cytokines promote insulin resistance via c-Jun terminal kinase activation. Mitochondrial ROS overproduction impairs functions by changing the redox balance. Accumulation of misfolded proteins (IAPP-diabetes, Aβ-Alzheimer’s disease) affects autophagy pathways. Hyperglycemia favors the formation of toxic nonenzymatically glycated proteins and lipids. Oxidative stress triggers inflammation, reduces cell survival, and promotes apoptosis. Abbreviations: O_2_•^−^, superoxide radical; NO_2_•, nitrogen dioxide radical; HO•, hydroxyl radical; NO•, nitric oxide radical; IL-6, interleukin 6; IL-12, interleukin 12; TNF-α, tumor necrosis factor alpha; TNFR1, TNF receptor 1; JNK, c-Jun N-terminal kinase; ^P^JNK, phosphorylated JNK; ^P^Ser, phosphorylated serine; IRS-1, insulin receptor substrate 1; AGEs, advanced glycation end products; Aβ, amyloid beta peptide; IAPP, islet amyloid polypeptide; ROS, reactive oxygen species; DNA, deoxyribonucleic acid.

Metabolic dysfunction and neurodegeneration are associated diseases, both showing a high prevalence in middle-aged or elderly people [1]. Accumulating evidence has suggested the presence of a strong correlation between abnormalities in glucose homeostasis and cognitive dysfunction [2]. Furthermore, inflammation and insulin resistance are considered an important mechanistic link between obesity, diabetes, and Alzheimer’s disease (AD)-related dementia [3,4,5]. Inflammation is a protective response to various stimuli that involves the discharge of harmful foreign compounds and adjustment of the damaged tissues. This process includes activation of innate immune cells and adaptive immune response, followed by release of numerous mediators to the site of assault [6]. Insulin resistance is one of the major hallmarks involved in the development of type 2 diabetes and is mainly associated with high blood sugar levels [7]. Data from recent years show that optimal insulin signaling homeostasis is important to the maintenance of brain health [8]. In addition, excessive free radical generation, known to promote oxidative stress and cytokine production, appears to be the main trigger factors for cellular dysfunction including impaired insulin signaling and mitochondrial dysfunction (Figure 1).

**Figure 2:**
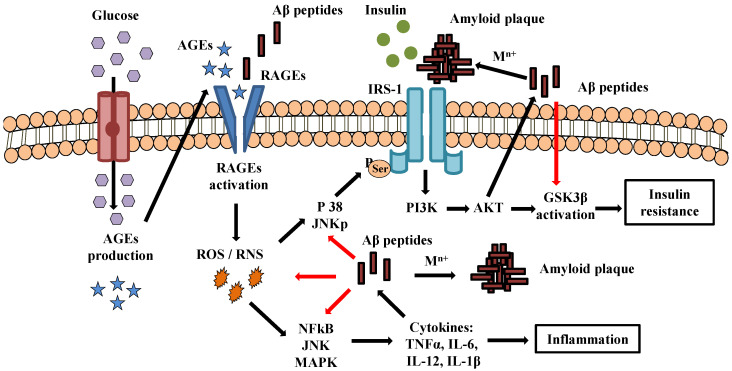
Proposed mechanism linking insulin resistance and inflammation with amyloid plaque formation. Hyperglycemia and inhibition of glycolysis favor accumulation of glucose and production of advanced glycation end-products (AGEs). The interaction between AGE-specific receptors and its ligands (RAGEs activation) increases oxidative stress generation (ROS/RNS) and turns on transcription of proinflammatory cytokines. Oxidative stress causes mitochondrial dysfunction which, in turn, promotes abnormal serine phosphorylation of IRS-1 (insulin receptor substrate protein) upon activation of phosphorylated JNK (c-Jun N-terminal kinase). The presence of toxic amyloid plaques degrades IRS and competes with insulin in IR activation. A decrease in insulin receptor signaling leads to inhibition of AKT (protein kinase B) and dephosphorylation (activation) of GSK-3β (glycogen synthase kinase 3 beta) while promoting generation of Aβ peptides under insulin-resistant conditions. Aβ peptides promote the development of ROS, mitochondrial dysfunction, and pro-inflammatory products, thereby indirectly further promoting insulin resistance. The formation of toxic amyloid plaque is caused by abnormal interaction of Aβ peptides with metal ions such as Zn^2+^, Cu^2+^, and Fe^3+^. Abbreviations: IL-6, interleukin 6; IL-12, interleukin 12; TNF-α, tumor necrosis factor alpha; IL-1β, interleukin 1β; Aβ, amyloid beta peptide; PI3K, phosphoinositide 3-kinase; NF-kB, nuclear factor kappa B; MAPK, mitogen-activated protein kinase; ROS, reactive oxygen species; RNS, reactive nitrogen species.

Accumulation of amyloid-like aggregates is a key pathological hallmark across a series of neurodegenerative diseases including AD and Parkinson’s disease. These amyloid fibrils are defined by several properties including a cross-β structure [9], insolubility in ionic detergent, protease resistance, and recognition by diagnostic indicator dyes [10]. In AD, amyloid beta peptide (Aβ)1-40/42 is the main component of amyloid plaques. According to the ‘amyloid hypothesis’, the disease is triggered by abnormal generation and/or clearance of neurotoxic Aβ. The hydrophobic peptide is secreted in the extracellular space after amyloid precursor protein (APP) proteolytic cleavage and distributed in various forms such as monomers, dimers, oligomers, protofibrils, and ultimately fibrils accumulation leads to formation of senile plaques [11]. It has been suggested that Aβ peptide causes activation of microglia and astrocytes leading to the release of cytotoxic molecules via proinflammatory cascade [12]. In addition, exposure to bacterial amyloid may influence the immune system, thereby altering brain Aβ homeostasis by enhancing inflammatory responses to endogenous neuronal amyloids [13]. The possible mechanism linking insulin resistance and inflammation to amyloid plaque formation is described in Figure 2.

Another peptide that possesses amyloidogenic properties is the peptide hormone islet amyloid polypeptide (IAPP, Amylin). Amylin is synthesized and co-secreted along with insulin by the pancreatic β cells [14]. Amylin deposition contributes to insulin resistance and oxidative stress being responsible for the development of type II diabetes mellitus (T2DM) by forming toxic amyloid aggregates leading to β-cell mass loss and subsequent reduction in insulin secretion. Since it can cross the blood–brain barrier, amylin is present in several peripheral organs of patients with T2DM. Thus, hyperamylinic condition observed in the diabetic brain may explain the development of AD pathology in T2DM [15]. Subsequent research has shown that amylin plays an important role in the complex process of glucose homeostasis by reducing glucose uptake into the bloodstream via multiple mechanisms such as suppression of glucagon secretion, slowing gastric emptying, and increasing satiety through a centrally mediated mechanism [16]. However, in diabetic patients, amylin becomes dysregulated due to a loss of insulin signaling. For example, recent studies demonstrated that addition of insulin to amylin aggregates promotes amylin aggregation. In addition, insulin can also undergo aggregation in its partially unfolded state and form ordered amyloid fibrils such as insulin–amylin co-aggregates [17].

It is well-established that hyperglycemia and insulin resistance promote the development of T2DM. However, a deficit in insulin signaling was observed in the brains of AD patients, a phenomenon referred to as “brain diabetes” [18]. As such, inhibition of brain insulin signaling can cause impairment of memory and cognition. Mechanistically, the canonical insulin signaling pathways are involved in maintaining glucose homeostasis and cellular homeostasis through the phosphatidylinositol 3-kinase/protein kinase B (PI3K/Akt) pathway. For instance, glycogen synthase kinase (GSK)3β activity may be suppressed upon phosphorylation by Akt while promoting synthesis of intracellular glycogen [19]. However, under insulin resistance conditions, characterized by a reduced PI3K/Akt signaling, GSK3β presents inappropriate activation. Concomitantly, the mitogen-activated protein kinase (MAPK) signal transduction pathway influences the mitogenic activity having an important role in the production of pro-inflammatory cytokines. Furthermore, activation of toll-like receptors (TLRs), responsible for the innate immune response, is considered another relevant phenomenon underlying inflammation-induced insulin resistance [20]. Taken together, compounds capable of enhancing insulin signaling in the brain may provide positive effects in treating AD.

Hyperglycemia is also considered a major contributor to reactive oxidative species (ROS) production and associated damage in diabetes. Excessive ROS production impairs oxidative phosphorylation, thus favoring insulin resistance. Multiple studies showed the presence of oxidative markers in urine, plasma, and tissues of diabetic patients [21]. In hyperglycemic condition, the inflammatory response can be induced by the increase in mitochondrial activity which generates oxidative stress and enhances ROS production, leading to the activation of inflammatory pathways [22]. Another characteristic is the overproduction of proinflammatory cytokines due to hyperactivation of microglia and astrocytes, the immune cells of the brain [15]. Likewise, neurodegenerative diseases are characterized by an increase in cytokines and ROS production due to microglial and mitochondrial dysfunctions leading to harmful effects on nerve cells [23]. However, in as much as hyperglycemia and insulin resistance were found to promote neuronal apoptosis and cognitive impairment, it remains unclear whether brain mitochondrial dysfunction found in obesity is a primary cause of brain insulin resistance [24].

Given that insulin resistance is mainly identified by inactivation of insulin signaling receptors, treatments based on upregulation of serine/threonine protein kinase Akt/PKB may provide new pathways in the management of T2DM [25]. However, an abnormal phosphorylation of Akt substrates was observed in AD brain implying that therapeutics directed to activate the Akt pathway requires prudence in neurodegenerative diseases [26]. On the other hand, models of AD [27] and T2DM [28] showed significant improvements following downregulation of inflammatory responses. Recent data revealed that small bioactive compounds exhibit potential protective functions and may be considered as alternative resource for pharmaceutical drug design against insulin resistance-related disease, providing new direction in drug discovery. Therefore, the following sections highlight the potential use of small active compounds through their antioxidant and anti-inflammatory properties in ameliorating toxicity associated with amyloidosis and hyperglycemia. This may lead to potential sources of therapeutic strategy to prevent and/or treat diabetes and neurodegenerative disorders.

## 2. Small Bioactive Compounds

There is a growing interest in identification of novel therapeutics to broaden the pharmacological effects while minimizing dose-dependent toxicology in the treatment of neurodegenerative disorders [29]. Extensive research has focused on chemistry driven approaches by controlling the physicochemical properties of compounds and integration of traditional small molecules into a more complex structure. This led to the development of peptide-drug conjugates, considering the benefits of hybrid therapeutics relative to classical molecules [30].

The current pharmacological approaches for diabetes mellitus and neurodegenerative diseases are unable to reverse diseases and focus mainly on managing symptoms by slowing progression. Furthermore, most modern drugs cause several adverse effects exacerbating the medical conditions following prolonged treatment [31]. However, recent advancements in proteomics have made it possible to obtain high quality peptide–drug conjugates though a variety of coupling techniques. For example, a wide range of hybrid drugs with enhanced effects can be obtained based on a simple covalent binding of a small organic drug molecule to a peptide. Indeed, several small bioactive compounds possessing pharmacological features and a carboxyl functional group available for covalent interaction with the N-terminal amine group of a peptide, critical for solid phase peptide synthesis technique, have been identified. For the purpose of this review, we only focused on compounds exerting protective effects against toxicity associated with hyperglycemia and amyloid aggregates.

### 2.1. Ascorbic Acid

Ascorbic acid, also known as vitamin C, is an essential water-soluble vitamin found in citrus fruits and green vegetables [32]. This nonenzymatic antioxidant is highly concentrated in the brain and participates in several important hydroxylation reactions [33]. The antioxidant properties of ascorbate are due to its ability to donate electrons to neutralize free radicals. As a result, ascorbic acid was found to protect dopaminergic neurons against excitotoxicity [34], fighting bacterial infections and promoting detoxifying reactions [35]. Likewise, ascorbic acid can switch neuronal metabolism from glucose consumption to uptake and use of lactate as a metabolic substrate to sustain synaptic activity [36].

Current evidence examining the effects of vitamin C supplementation in improving blood glucose levels and glycosylated hemoglobin (HbA1c) is, at best, inconsistent. For example, two clinical studies conducted on approximately 30 patients with T2DM showed a decrease in postprandial blood glucose after 2 × 500 mg per day Vitamin C supplementation alone for 16 weeks [37] or in combination with metformin for 12 weeks [38]. Although the monotherapy trial was longer, no improvements in HbA1c and fasting blood glucose (FBG) levels were observed, compared to the combination therapy that decreased FBG and HbA1c levels in T2DM patients. These results are in line with those from a large-scale trial (12 months, 152 subjects) showing the therapeutic efficacy of ascorbate-metformin combination therapy (500 mg per day) in reducing FBG and HbA1c levels [39]. Similarly, oral supplementation of vitamins C (2 × 500 mg daily) together with metformin (2 × 500 mg daily) significantly alleviated the laboratory related indicators in T2DM male patients compared to the control group [40]. These findings suggest that ascorbate may have clinical significance in the management of metabolic syndrome only when administered in combination with other glucose-lowering drug compounds. It should be noted, however, that although some studies showed beneficial effects of ascorbic acid in lowering postprandial glucose, most studies are short term trials and include relatively small number of subjects. Thus, studies using larger sample sizes and longer supplementation period are required to confirm the beneficial effects and/or long-term side effects of vitamin C supplementation as well as its mechanisms of action.

Since inflammatory responses are known to accompany hyperglycemia, insulin resistance, and protein aggregation, several in vitro studies investigated the antioxidant activity of ascorbic acid in oxidative stress-related diseases. For example, administration of ascorbate to streptozotocin-induced diabetic rats, improved lipid peroxidation in the brain prefrontal cortex, as well as superoxide dismutase (SOD) content and catalase activity, suggesting that the antioxidant activity of vitamin C may help restoring neuronal integrity and viability [41].

The strong relation between oxidative stress, metabolic syndrome, and AD suggests that the antioxidant systems may have an important role in the prevention and control of neurological outcomes in AD. In this regard, various in vitro studies using human neuroblastoma cell line (SH-SY5Y), a model for neurodegenerative disorders, revealed that ascorbic acid was able to suppress human insulin aggregation [42] and prevented apoptosis induced by Aβ fibrillation [43,44] providing great therapeutic potential for amyloid disorders. Similar results were obtained with ex vivo cultivated murine pancreatic cells, RIN-m5f β, where ascorbic acid increased cell viability by offering protection against amylin toxic fibrils [45]. Finally, preclinical research using APP/PSEN1 mice as models of AD revealed that low levels of brain ascorbic acid increases oxidative stress in the brain, mortality, and the risk for epilepsy and seizures [46]. Taken together, these findings suggest that ascorbic acid possesses the ability to reduce insulin impairment and defend cells against toxicity associated with amyloid fibrils while binding to the partially unfolded monomers, thus preventing them from undergoing conformational changes that may lead to toxic amyloid fibril formation. Notwithstanding its known strong free radical scavenging properties, the results from clinical trials evaluating the effects of ascorbic acid are conflicting thus limiting its use as therapeutic agent.

### 2.2. Acetylsalicylic Acid

Acetylsalicylic acid (ASA), also known as aspirin, is a common non-steroidal antiinflammatory drug (NSAID) used for the treatment of pain, reduce inflammation and fever due to various causes [47]. The mechanism of action involves irreversible inactivation of both cyclo-oxygenase COX-1 and COX-2 that leads to inhibition of prostaglandin (PG) and thromboxane synthesis [48]. Additional effects, besides preventing platelet aggregation, include modulation of nuclear factor kappa-light-chain-enhancer of activated B cells (NF-κB) pathway, downregulation of inducible nitric oxide synthase (iNOS), oxidative phosphorylation uncoupling, and increased permeability in mitochondria [49].

The ability of aspirin to specifically inhibit the protein inhibitor of kappa light polypeptide gene enhancer in B-cells, kinase beta (IKK-β) and further regulate NF-κB signaling pathway results in attenuated insulin resistance while promoting insulin sensitivity. For instance, in mice fed for 3 months with ASA-supplemented diet (30 mg/kg), ASA reversed hyperinsulinemia and hyperglycemia bringing insulin and glucose to normal levels after 2 months of treatment [50]. In a similar study, insulin resistance in streptozotocin (STZ)-induced type 2 diabetic rats was significantly improved by inhibiting hepatic NF-κB activation and tumor necrosis factor-α (TNF-α) level after two month of aspirin administration (120 mg/kg/day) [51]. ASA decreased glucose blood levels and increased serum arginine vasopressin hormone levels after 5 weeks of oral treatment (150 mg/kg/day) in STZ-induced diabetic mice [52]. Furthermore, in vivo data suggest that a dose of 30 mg/kg of ASA was able to prevent oxidative damages caused by an intraperitoneal injection of peroxide hydrogen in mice and established the redox homeostasis [53]. Moreover, recent studies also showed that oral administration of Zn(ASA)_2_ complex to Zucker diabetic fatty rats reduced plasma glucose-levels and prevented diabetic cardiomyopathy [54]. Although preclinical studies using animal models suggest that aspirin can interfere in the development of insulin resistance, the results from human clinical trials are less convincing, with ASA alone having a lower effect on metabolic damage than combined therapy. For example, administration of aspirin combined with common diabetes medications potentiated their antioxidant effects and reduced oxidative stress [55]. Furthermore, the increased risk of gastrointestinal bleeding following administration of aspirin limits its use as long-term pharmacotherapy [56].

The existence of a link between platelets over activation and amyloid precursor protein expression [57] suggest that antiplatelet drugs, such as ASA, that are capable of inhibiting NF-κB pathway might have beneficial effects on reducing Aβ deposition in the Alzheimer brain [58]. This theory was confirmed in a recent study that used non-selective, NSAIDs drugs, such as aspirin and sodium salicylate, to evaluate memory impairments and Aβ-mediated suppression of synaptic plasticity in the hippocampus of AD model rats [59]. The results showed that sub-chronic high dose of sodium salicylate and chronic low dose of aspirin improved synaptic dysfunction underlying memory deficits via a COX-dependent mechanism. Similarly, oral administration of low-dose of aspirin decreased amyloid plaque pathology in AD mice in a peroxisome proliferator-activated receptor alpha (PPARα)-dependent manner [60]. Furthermore, the inhibitory effect of ASA was found to affect insulin fibrillation and increase cellular viability in a model of insulin mediated toxicity in human breast cancer cells [61]. While animal studies showed beneficial effects of NSAID treatment in AD, inconsistent results have been reported following clinical trials. Hence, beside the anti-inflammatory and ROS-suppressive effects of aspirin, the use of aspirin for glycemic control in diabetics is controversial and the associated risks of developing bleeding events following their administration should be taken into consideration.

### 2.3. Gallic Acid

Gallic acid (GA) or 3,4,5-trihydroxicbenzoic acid is an endogenous plant polyphenol found abundantly in tea, grapes, berries, and other fruits as well as in wines. Derivatives of gallic acid were shown to provide a wide range of effects such as antimicrobial, antioxidant, gastroprotective, neuroprotective, cardioprotective, anticancer, and anti-inflammatory activities [62]. For example, treatment with gallic acid (GA, 20 mg/kg) administered for six weeks significantly ameliorated glucose tolerance, and diminished brain oxidative stress and inflammation in STZ-induced diabetic rats while reducing TNF-α serum levels [63]. It also had a potent anti-hyperglycemic and antihyperlipidemic effect in diabetic rats through increasing the levels of adiponectin and peroxisome proliferator-activated receptor gama (PPARγ) mRNA [64]. Orally administered GA (20 mg/kg) for 45 days reduced blood glucose levels while increasing insulin concentration and protecting the integrity of erythrocyte membrane in alloxan-induced diabetes in Wistar rats [65]. Besides monotherapy, the GA generated antihyperglycemic activity in combination drug therapy. For example, a combination ratio of 20:10 mg/kg gallic acid: andrographolide, significantly improved glucose tolerance in STZ-induced diabetic rats [66]. Of interest is the fact that GA has inhibitory potential against insulin aggregation only after undergoing oxidation. Thus, its oxidized form inhibits primary nuclei formation and alters insulin amyloid aggregation pathway [67].

Spectroscopic results also revealed that GA constrains the conformational transition of α-helix to β-sheet, associated with insulin fibril formation, and inhibits insulin aggregation in acidic pH [68]. In addition, GA was found to express anti-fibrillogenic activity against Aβ40 amyloid fibril, carboxymethylated kappa-casein [69] and hen egg white lysozyme [70], compounds used as models of amyloid fibril formation. Finally, GA administration to a transgenic *D. melanogaster* model of AD decreased activity of cholinesterases and β-secretase, known to play a critical role in AD pathophysiology [71]. Regarding its metal-chelating activity, a recent study showed that GA was able to inhibit formation of metal-induced aggregates suggesting a bi-functional anti-aggregation property for GA [72]. Overall, the benefits of GA are related to its anti-fibrillogenic as well as antioxidant effects, characteristics required for any drug molecule used in prevention or control of these diseases.

### 2.4. Protocatechuic Acid

Protocatechuic acid (3,4-dihydroxybenzoic acid, PCA) is a phenolic acid found in a large variety of plants and other natural resources. This bioactive compound possesses many pharmacological activities such as antioxidant, antibacterial, anticancer, antiulcer, antidiabetic, antiaging, antiviral, anti-inflammatory, and neuroprotective effects. For instance, orally administrated PCA to STZ-diabetic rats reduced glucose plasma and glycosylated hemoglobin levels and increased insulin and hemoglobin production [73,74]. Similarly, PCA stimulates insulin signaling pathway by increasing glucose transporter type 4 (GLUT4) translocation and glucose uptake in human adipocytes [75,76]. Furthermore, in silico findings showed that PCA supports the interactions with different signaling molecules involved in glucose metabolism and inflammatory pathways, in agreement with previous in vitro and in vivo studies [77]. For example, PCA downregulates phosphorylation of p38 MAPK in high glucose-induced human mesangial cells [78]. In combination with cyanidin-3-*O*-βglucoside, PCA activated AMP-activated protein kinase (AMPK) and downregulated mechanistic target of rapamycin/S6 kinase (mTOR/S6K) pathway both in vitro and in vivo systems in order to improve glucose homeostasis and increase insulin sensitivity [79]. At the same time, various studies evaluating the anti-inflammatory activity of PCA showed that phenolic acid can augment the antioxidant status [80,81,82], inhibit lipid peroxidation [83], and suppress several pro-inflammatory biomarkers [84,85].

Another advantageous feature of PCA is the neuroprotective effect exerted by downregulation of receptor activator of NF-κB ligand-induced inflammatory proteins, inhibition of MAPK activation, and reduction of NF-κB expression [86]. As such, diet rich in PCA showed in vivo and in vitro neuroprotective effects in transgenic mouse model for AD [87] and C6 glial cell. Similarly, a significant decrease in lipid peroxidation, nitric oxide production and neuroinflammation was also observed in Aβ25-35-injected mice after PCA administration [88,89]. This resulted in cognitive deficits improvement, reduction in Aβ deposition and decreased inflammatory responses in aged AβPP/PS1 double transgenic AD-model mice treated with PCA [90]. In addition to Aβ fibril destabilization, PCA treatment was found to prevent death on pheochromocytoma PC12 cells and inhibit αS proteins aggregation known to induce Lewy bodies in the brain [91]. The protective effects of PCA were also confirmed in recent studies, demonstrating an increase in Akt/GSK-3β/myocyte enhancer factor 2D (MEF2D) [92] and decrease in P38/JNK-NF-κB signaling pathway activity [93]. In turn, in BV2 microglial cells, PCA mediated the anti-neuroinflammatory effects by suppressing toll-like receptor 4 (TLR4)-mediated NF-κB and MAPKs activation [94] via regulation of sitruin 1 (SIRT1)/NF-κB pathway [95]. All together, these findings indicate that PCA exerts protective effects associated with attenuation of glycemic status, inflammation responses, and amyloid deposits.

### 2.5. p-Coumaric Acid

p-Coumaric acid (p-CA) or 4-hydroxycinnamic acid (p-HCA) is a compound found in plants that features numerous bioactivities such as immunomodulatory, anti-inflammatory, antioxidant and neuroprotective effects [96]. In addition, p-CA reduces peroxidation of low-density lipoproteins and inhibits cellular melanogenesis [97]. The antihyperlipidemic and antidiabetic effects of p-HCA were confirmed in STZ-induced diabetic rats demonstrating an increase in high density lipoprotein (HDL), hemoglobin and plasma insulin, associated with lowered blood glucose, glycosylated hemoglobin, and lipid profiles [98].

Several studies indicate that p-CA has a potent antioxidant, anti-inflammatory, and anti-apoptotic impact on diabetic-induced dysfunctions. For example, treatment of diabetic rats with p-CA enhanced the activity of antioxidant enzymes, attenuated brain oxidative stress and regulated apoptosis through B-cell lymphoma 2 (bcl-2)/bcl-2 associated X (bax) proteins expression [63]. Other studies revealed a reduction in glucose transporter Glut-2 expression following p-CA administration to diabetic rats suggesting an improvement in glucose homeostasis [99]. Besides its hypoglycemic effect, p-CA was found to attenuate oxidative stress via regulation of SOD and malondialdehyde levels, mediate inflammation by targeting TLR-4 and interleukin (IL-6) and diminish renal fibrosis mediated by transforming growth factor β1 (TGFβ1) [100]. Furthermore, its antidiabetic activity may be mediated via modulation of TNF-α and adipocytokines secretions and upregulation of PPARγ mRNA expression [64].

When examining the bioactivity of p-HCA in the brain, in vitro study on primary cortical neurons revealed that p-CA has a potent inhibitory effect against 5-S-cysteinyl-dopamine induced neurotoxicity and thus, counteracts the progression of neurological disorders [101]. The neuroprotective activity of p-CA was also confirmed in pheochromocytoma PC12 cells where it suppressed formation of ROS [102], attenuated beta amyloid-induced apoptosis [103,104], and inhibited expression of inflammatory target proteins via inactivation of NF-κB and MAPKs signaling pathways [105]. Recent in vivo and in vitro experiments showed that p-CA promoted neural stem cell proliferation via activation of brain-derived neurotrophic factor (BDNF)/tropomyosin receptor kinase B (TrkB)/AKT signaling pathway, improved spatial learning and memory functions, and reduced anxiety in post–ischemic stroke rats [106]. Thus, the beneficial effects of p-coumaric acid are mainly related to its ability of inducing BDNF-TrkB signaling regulation, mediate expression of apoptotic factors such as TLR4, IL-6, TGFβ1 and inhibiting amyloid fibrillogenesis.

### 2.6. Ferulic Acid

Ferulic acid (4-hydroxy-3-methoxycinnamic acid, FA) is a phenolic acid that has low toxicity and exhibits a wide range of physiological functions such as antioxidant, antimicrobial, anti-inflammatory, and anti-cancer activities [107,108]. In addition, several studies showed that FA inhibits expression and/or activity of cytotoxic enzymes, thus providing potential treatment for many disorders including AD, cancer, cardiovascular diseases, diabetes mellitus, and skin disorders [109].

When evaluating the antidiabetic property, FA not only improves hepatic glycogenesis by phosphorylation and inhibition of GSK-3β but also reduces hepatic glucose production by preventing the interaction between forkhead box protein O1 (FoxO1) and the genes involved in gluconeogenesis [110]. These findings suggest that FA attenuates cognitive impairment in rat model of diabetes mellitus by regulating protein-tyrosine phosphatase 1B and insulin signaling pathway [111]. In addition to monotherapy, insulin-FA combination therapy improved peripheral nerve function in diabetic rats via enhancing glycemic control and suppressing inflammatory and apoptotic markers [112]. When tested in high fat-induced obesity, FA treatment decreased hepatic lipogenesis and gluconeogenesis [113,114,115,116]. Finally, FA administration alleviates insulin resistance [117,118] and inhibits insulin fibrillation associated with T2DM [119].

The neuroprotective characteristics of FA were evaluated in various studies. For instance, in vitro experiments showed that FA was able to interact with Aβ peptide in the initial stage of the aggregation process and redirected the peptide self-assembly to the formation of nonfibrillar amorphous aggregates [120,121,122,123]. Similar results were observed in in vivo studies using long-term administration of FA for treating Aβ toxicity. In this case, a significant decreased in cortical IL-1β levels and amyloid deposition was observed in the brain of AD model rats [124]. Likewise, FA administration to AD-like rats model attenuated performance impairment and memory deficits induced by Aβ deposits [125,126]. In a recent research using *C. elegans* AD model worms, FA ameliorated Aβ-induced pathological symptoms by the autophagy pathway via a fasting-like effect [127]. Although most of these studies are in animal models or in vitro, they are promising by demonstrating that ferulic acid may be able to effectively fight against neurodegenerative diseases caused by protein misfolding or pathological aggregation while exerting protective effects against insulin impairments.

### 2.7. Sinapic Acid

Sinapic acid (3,5-dimethoxy-4-hydroxycinnamic acid, SA) is a cinnamic acid derivative that presents antioxidant, antimicrobial, anti-inflammatory, and anticancer activity being suggested for potential use in food processing, cosmetics and pharmaceutical industry [128]. In this regard, experiments evaluating its anti-inflammatory effects have revealed that SA inhibits production of iNOS, COX-2, and proinflammatory cytokines via suppression of NF-kB activity in macrophages [129]. Moreover, based on its toxicological behavior, SA possesses free radical scavenging activity as well as chelating properties [130].

SA has been shown to exert antidiabetic and antioxidant effects. For example, administration of SA to STZ-induced diabetic rats reduced hyperglycemia via phospholipase C/protein kinase C (PLC/PKC) [131] and delayed progression of diabetic nephropathy via nuclear factor erythroid 2-related factor 2 (Nrf2)/heme oxygenase-1(HO-1) mediated pathways while upregulating the antioxidant defense enzyme activities [132]. Similarly, SA administration suppressed serum endothelin 1, IL-1β contents, and expression of pyroptotic proteins via downregulation of long noncoding RNA (lncRNA), metastasis-associated lung adenocarcinoma transcript 1 (MALAT1) in diabetic atherosclerosis rats [133]. Furthermore, a significant reduction in malondialdehyde and ROS levels in liver and colon of high-fat diet (HFD) rats highlighted the antioxidant ability of SA [134]. In 3T3-L1 adipocytes cells, the phenolic treatment was able to upregulate proteins involved in mitochondrial thermogenesis causing fats oxidation via the p38 MAPK/cyclic AMP response element-binding protein (CREB) signaling pathway [135]. Nevertheless, the importance of PKA/p38-mediated pathway and CREB signaling in thermogenic lipolysis were recently observed in a study that used SA as activation agent for brown adipocyte [136].

The protective effects of SA were also exerted against neuronal damage induced by Aβ protein deposition. For example, amelioration in memory impairment and neuronal cell death along with attenuation of glial activation and tyrosine nitration were observed following SA administration to mouse model of Aβ1-42 protein-induced AD [137]. Likewise, an improvement in antioxidant status followed by prevention of cognitive impairment was noticed after SA administration to intracerebroventricle-STZ-induced Alzheimer rats. In this case, SA reduced the number of caspase-3 positive cells, significantly attenuated astrocyte activation by lowering the levels of TNF-α, and diminished the influence of amyloid peptide on iNOS expression levels while decreasing the neurotrophin-3 expression in the hippocampal CA1 region [138]. Recently, new insights into the neuroprotective role of SA suggested that the phenolic treatment possesses not only free radical scavenging and anti-inflammatory properties but also has an important role in decreasing expression of choline acetyltransferase and neuronal loss [139]. In summary, results thus far show that SA holds therapeutic potential in multifactorial disease since it can target multiple mechanistic pathways—such as oxidative stress, inflammation, and cholinergic dysfunction.

### 2.8. Lipoic Acid

Lipoic acid (LA, 1,2-dithiolane-3-pentanoic acid), also known as thioctic acid, is a naturally occurring compound that serves vital functions at the cellular level. LA and its reduced form dihydrolipoic acid create a potent redox couple that exists endogenously in tissues and acts as a cofactor for some mitochondrial enzymes such as pyruvate dehydrogenase and alpha-ketoglutarate dehydrogenase [140]. Interestingly, LA stimulates glucose oxidation without influencing glycolysis and lactate or palmitate oxidation process [141].

Given its potential to safely enhance insulin sensitivity, the possible health benefits of LA supplementation have been tested in hyperglycemic conditions. Previous studies showed that LA treatment is able to increase insulin-stimulated glucose metabolism [142] and glutathione levels in tissue [143] in T2DM. Since oxidative stress can cause renal dysfunction, the interactions of LA, and its efficacy have been tested in the prevention of oxidant damage in diabetic rats. Hence, a clear reduction in oxidative stress by regulating the overexpression of NADPH oxidase [144] along with restoring the activity of antioxidant enzyme superoxide dismutase [145] was noticed in diabetic kidneys after LA administration. Similar findings showed a decrease in inflammatory pathway proteins and oxidative stress inhibition after LA treatment in HFD-induced cerebral damage mice [146] and diabetic patients [147]. Chemically, LA possesses a chiral center and although the majority of the commercial product consists of a racemic admixture, the R form is the biologically active form produced by the body while the S form is an inactive chemical product. In a recent study, Ghelani et al. found that (R)-α-lipoic acid manifested protective effect on low-dose STZ-injected and HFD-fed model rat of metabolic disorders by strongly decreasing the hyperglycemic and hyperlipidemic events [148]. Interestingly, a DL-α-lipoic acid was found to protect the 3-mercaptopyruvate sulfurtransferase/H_2_S pathway against hyperglycemic-induced impairment by restoring the ability of 3-mercaptopyruvate to induce cellular bioenergetics, angiogenesis, and wound healing following in vivo administration [149]. Although LA supplementation produces many beneficial effects such as improvement of nuclear erythroid-related factor 2 activity, promotion of fatty acid oxidation, reduction of visceral adipose tissue, and regulatory T cells conservation [150], long-term administration of LA may trigger autoimmune hypoglycemia in people with genetic predisposition [151].

Given that LA possesses a strong antioxidant character, several studies examined its neuroprotective effects in ameliorating the development of AD. For instance, LA may protect neurons against cytotoxicity induced by Aβ peptide through inhibition of NFkB activation and scavenge free radicals [152] as well as PKB/Akt signaling pathway [153]. In addition to direct antioxidant effects, LA/DHLA (dihydrolipoic acid) redox couple generated neuroprotective effect against Aβ and inhibited fibrils formation in a dose-dependent manner by covalent binding to lysine residues of Aβ [154]. Furthermore, combinations therapy containing α-lipoic acid diminished β-secretase activity, enhanced neprilysin activity [155], reduced amyloid beta accumulation, and decreased memory deficits [156] in aged rat brain. Other in vivo experiments revealed that LA supplementation can restore hypoxia-inducible factor-1a (HIF-1α) expression and alleviate cognitive deficits by reinstating glucose metabolism impairment via the BDNF/TrkB/HIF-1α signaling pathway [157] while combination of exercise training and α-lipoic acid treatment improved the antioxidant status in NSE/APPsw-transgenic mice [158]. Likewise, in vitro studies showed that LA protects against H_2_O_2_-induced cell death [159] and buthionine sulfoximine [160] via activation of PI3K-Akt pathway. It is also important to note that LA exerts chelating properties and offer protection against aluminum toxicity [161], cadmium lethality [162], and copper dysregulation [163]. Together, these studies show that the bio-thiol antioxidants LA and its active metabolite DHLA incorporate several impressive features vital for cellular protection such as metal-chelating activity, ROS scavenger, antioxidant regenerator, and destabilization potential on amyloid fibrils.

### 2.9. Rosmarinic Acid

Rosmarinic acid (RA) is a natural widespread caffeic acid ester widely identified in different plant species commonly used as culinary herbs [164]. This hydroxylated compound has received significant attention for its antibacterial, anti-inflammatory, anti-aging, anticancer, antidiabetic, cardioprotective, hepatoprotective, nephroprotective, antidepressant, antiallergic, and antiviral properties [165]. Due to both its antioxidant and antimicrobial properties, RA is used in pharmaceutical and cosmetic industries as a food preservative [166].

Numerous studies have examined the anti-diabetic effects of rosmarinic acid using in vivo experiments in streptozotocin treated rats. For example, administration of RA to STZ-induced diabetic rats improved insulin sensitivity and glucose uptake by attenuating gluconeogenesis and modulating glucose uptake through AMPK pathway [167]. Likewise RA administration led to a significant reduction in oxidative stress and glucolipotoxicity, increase in SOD activity, and reduced oxidative stress damage by preventing lipid peroxidation and increasing acetylcholinesterase activity in diabetic animals [168,169,170]. Furthermore, this phenolic acid diminished activity of hepatospecific pathophysiological enzymes, restored activities of key carbohydrate metabolizing enzymes and improved hepatic glycogen content in a similar animal model [171]. In addition to its significant antioxidant property, RA improved glucose homeostasis and insulin sensitivity by decreasing expression of gluconeogenic enzyme phosphoenolpyruvate carboxykinase (PEPCK) and increasing GLUT4 levels in skeletal muscle [172]. These findings have been corroborated by Jayanthy et.al. showing that RA treatment augmented insulin signaling and mitochondrial biogenesis via increasing GLUT 4 translocation and AMPK activation [173]. Interestingly, RA was found to inhibit insulin’s [174] and amylin’s [175,176] ability to form amyloid fibrils, suggesting new avenues in its pharmacological applications.

It is well known that oxidative stress plays a key role in the progression of various neurodegenerative diseases. Thus, several studies evaluated the neuroprotective effect of RA enriched extracts against Aβ-induced toxicity. Indeed, rosmarinic acid was found to offer protection against Aβ-induced toxicity in both in vitro [177] and in vivo [178] models of neurodegeneration. Similar results were obtained in a recent study showing that, in addition to its inhibitory effects on Aβ and human islet amyloid polypeptide (hIAPP) aggregation, RA can also prevent cell destruction related to AD and T2DM [179]. Mechanistically, administration of RA to Aβ-challenged PC12 cells mediated neuroprotection via Akt/GSK-3β/Fyn-mediated Nrf2 activation [180]. In vitro studies using SH-SY5Y neuroblastoma cells showed that RA significantly attenuated H_2_O_2_-induced ROS generation by stimulating the antioxidant enzyme HO-1 [181]. Furthermore, RA was able to suppress Aβ accumulation via dopamine-signaling pathway enhancement [182] and reduce inflammation by downregulating JNK signaling pathway [183]. Recent findings showed that RA interferes with Cu(II)-Aβ complex and forms original ternary association that modifies Aβ reactivity in terms of aggregation and ROS generation [184]. As expected, RA treatment promoted cognitive improvement and neuroprotection against Aβ25-35 [185], Aβ42 [186,187], and scopolamine [188] induced toxicity in rat model of AD. Similar results were obtained with other proteins such as serum amyloid A [189], Tau [190], or α-synuclein [191] that can incur protein misfolding and subsequent disease processes. This suggests that RA has an extended protective activity that can be utilized in drug development applications used for preventing and treating various metabolic and degenerative disorders.

### 2.10. Folic Acid

Folic acid (FoA) is the oxidized form of vitamin B9. Dietary FoA supplements are indicated for prevention and treatment of folic acid deficiency states such as megaloblastic or nutritional-deficiency anemia [192]. Vital for the formation of new cells, healthy growth, and normal fetal development, FoA is involved in DNA and RNA metabolism [193].

Several studies showed that FoA can ameliorate hyperglycemia and insulin sensitivity by altering mitochondrial dysfunction while exerting antioxidant activities [194]. For example, FoA administration reduced plasma homocysteine, improved glycemic control and insulin resistance in patients with T2DM [195]. Interestingly, an improvement in cholesterol profile and glucose metabolism by restoration of AMPK activation (phosphorylation) was seen in HFD fed mice after FoA administration [196]. To this end, FoA improved insulin resistance, reduced fat mass, and serum glucose in obese mice while exerting anti-inflammatory effects by lowering cytokine levels [197]. Supplementation of FoA had a positive effect on diabetes-associated oxidative stress. For example, a significant reduction in glucose levels, catalase, SOD antioxidant enzymes and malate dehydrogenase activities have been noticed in diabetic rats after FoA administration [198]. In addition, FoA treatment normalized the distribution of CuZnSOD protein and Bax/Bcl-2 ratio while lowering apoptotic rate and stimulating Vegf-A gene expression in the yolk sac of embryos of diabetic rats [199]. Similarly, daily administration of FoA to T2DM patients decreased DNA and oxidative damages by suppressing micronuclei, 8-OHdG, and lipid peroxides [200].

Several in vivo and in vitro experiments revealed that FoA decreases Aβ-peptide production. When patients with AD were treated for six months with FoA, there was a significantly lower expression of Aβ40 and TNFα-mRNA and higher Aβ42/Aβ40 ratio suggesting FoA’s role in inflammation coupled with lower cognitive decline compared to control group [201]. Similarly, a decrease in Aβ production via modulating DNA methyltransferase (DNMT) activity was observed in neuroblastoma N2a-APP cells exposed to FA [202]. In addition to its inhibitory effect, FoA was shown to protect neurons against amyloid toxicity. Indeed, an increase in cell viability and mitochondrial membrane potential was observed in Aβ 31-35-treated neurons after FoA administration [203,204]. FoA blocked Aβ oligomer-induced neuronal toxicity and increased cell viability by stimulating methylation potential and DNMT activity, decreasing presenilin 1 (PS1) and APP expression, thus altering promoter methylation in AD transgenic mice [205]. Studies conducted on APP/PS1 mice, a model of AD, revealed that FoA deficiency intensify Aβ generation improving APP and beta-site amyloid precursor protein cleaving enzyme 1 (BACE1) expression [206,207,208], suggesting that folic acid deficiency exacerbates cognitive impairment. Additionally, experimental and theoretical evidence showed that FoA generates suppressing effects on Tau fibril formation through a spontaneous hydrophobic interaction leading to native state stabilization of tau followed by a decline in protein–protein interactions and Tau oligomerization [209]. In combination therapy with compounds such as docosahexaenoic acid [210], memantine [211], α-Tocopherol [212], and isoflavones [213], FoA was able to enhance cognitive improvement while preventing the pathological alterations caused by Aβ toxicity. The above results collectively suggest that FoA exerts numerous beneficial effects, being responsible for restoring antioxidant enzymes activity, offering protective effects against insulin impairment, and inhibiting amyloid fibrillization.

### 2.11. Oleanolic Acid

Oleanolic acid or oleanic acid (OA, 3β-hydroxyolean-12-en-28-oic acid) is a naturally occurring pentacyclic triterpenoid widely distributed in food and plants known for its biological and pharmacological significance [214]. Besides its hepatoprotective property, OA can generate diverse biological activities, including anticancer, anti-osteoporosis, anti-obesity, anti-diabetic, anti-inflammatory, immune-regulatory, and antioxidant effects [215].

A number of studies have reported the protective activities of oleanolic acid against metabolic disease such as diabetes mellitus in different in vitro and in vivo models [216]. For example, in T2DM model, OA administration was found to reverse hyperglycemia and normalize blood glucose levels [217,218], improve hepatic insulin resistance [219], and suppress hepatic glucose production via the Akt/FoxO1 pathways [220]. Additionally, OA decreases blood glucose and plasma lipids while it significantly elevates plasma leptin and decreases ghrelin secretion in HFD-induced obese mice [221]. Interestingly, OA was found to lower glucose levels and enhance insulin signaling by mediating acetylcholinesterase release known to be responsible for muscarinic M3 receptors stimulation and insulin release [222]. In the liver, OA improved hepatic insulin resistance through inhibition of mitochondrial oxidative stress, hypolipidemic effect and decreasing inflammation via remarkable stabilization of Nrf2 and glutamate-cysteine ligase catalytic subunit (GCLc) expression [219]. Studies examining OA’s antidiabetic effects showed improved glycemic control [223] through inhibition of α-glucosidase activity [224], and reduced oxidative stress and antioxidant activity [225,226,227]. In addition to monotherapy, OA generated synergistic and complimentary actions in combined therapy with other antidiabetic drugs such as metformin and insulin. For instance, OA-metformin combination therapy significantly reduced blood glucose and insulin levels and improved liver pathology in diabetic mice [228]. Likewise, OA in synergy with 4IU insulin enhanced activation of the insulin signaling pathway and increased insulin-stimulated hypoglycemic activity [229].

Several studies examined the neuroprotective effects of OA in various models [230]. In pheochromocytom PC12 cells, OA improved catalase and SOD activities, attenuated release of IL-6 and TNF-α and decreased malondialdehyde formation [231]. When OA extracts from *Aralia cordata* were added to cultured rat cortical neurons, it inhibited neuronal death, glutamate release, and generation of ROS induced by Aβ-peptide(25–35) [232]. In mouse models, OA administration improved memory loss, alleviated neuronal damage and synapse changes in Aβ25-35-induced AD mouse models [233]. Similar results were observed against 6-hydroxydopamine induced toxicity in PC12 cells [231,234], parkinsonian rat model [235], and lipopolysaccharide-induced inflammation on mouse neuroglia BV2 cell line [236]. Recently, OA was shown to improve scopolamine-induced memory impairment through BDNF-ERK1/2-CREB pathway via TrkB activation [237] while inflammatory responses in human umbilical vein endothelial (HUVE) cells were reduced through the inhibition of high-mobility group box 1 signaling pathway [238]. Other protective mechanisms of OA against Aβ-induced neurotoxicity involve suppression of secretory phospholipase A2 group IIa mediated-calcium signals pathway in astrocytes [239], deregulated Janus kinase/signal transducers and activators of transcription (JAK/STAT) signaling [240], expression of stanniocalcin-1 glycoprotein [241], and expression of BDNF, TrkB, and CREB proteins [233]. Interestingly, in a recent in vivo experiment using transgenic APP/PS1 AD mouse model, OA attenuated cognitive deficits by stimulating the Wingless/integrated (Wnt)/GSK-3β/β-catenin pathway [242]. These studies show that OA has significant antioxidative, anti-inflammatory, and neuroprotective activities, and can ameliorate insulin sensitivity and inhibit apoptosis. Although OA does not exert significant disaggregating activities against amyloid fibrils, it possesses significant protective characteristics to be considered in future pharmacological applications.

### 2.12. Ursolic Acid

Ursolic acid (3β-3-hydroxy-urs-12-ene-28-oic-acid, UA) is a natural triterpene compound found in vegetables, medicinal plants, and common herbs that are known for their extended pharmacological properties. Considering its significant activity exerted in different systems, the pharmacology of UA has been extensively reported in recent years [243]. For instance, UA was shown to possess biological effects such as anti-inflammatory, antioxidant, antifungal, and antibacterial [244]. UA also exhibits antidiabetic functions by limiting the activity of pancreatic α-amylase, succinate dehydrogenase, glucose-6-phosphatase, and aldose reductase in non-obese T2DM mice [245]. It was recently reported that, UA treatment suppressed glucose-induced proliferation of mesangial cells and inhibited ROS generation and oxidative stress by restricting PI3K/Akt/mTOR pathway activation [246]. These findings are in agreement with previous research suggesting improvement in mesangial cells hypertrophy and proliferation under high glucose conditions [247]. Moreover, co-treatment of UA with Rosiglitazone, an antidiabetic, insulin sensitizer drug, suppressed lipid accumulation in liver, and diminished hepatic marker enzyme activities in high fat diet-fed C57BL/6J mice [248].

In addition to its anti-inflammatory effects, recent studies showed that UA improves insulin resistance and hyperinsulinemia in diet-induced obesity rats [249,250,251]. Indeed, UA administration decreased body weights and blood glucose via angiotensin II type 1 receptor (AT1R)-associated protein 1 (ARAP1)/AT1R pathway in db/db mice [252]. Likewise, UA reduced expression of inflammatory markers and improved activity of antioxidant enzymes in STZ-induced diabetic rats [253]. When used as a dietary supplement, UA restored adipokine/cytokine dysregulation, ROS production, and extracellular matrix accumulation in the liver, thereby avoiding hepatic lipotoxicity and fibrosis [254]. This resulted in a significant decrease of obesity risks and proinflammatory adipokines concomitant with increased adiponectin levels [255].

Finally, UA offered protection against toxicity associated with amyloid-beta peptide, an important mediator of AD progression. Using mammalian CHO cells expressing human CD36 to quantify Aβ binding, it was shown that UA obstructed Aβ-receptor CD36 interaction in a dose-dependent manner and blocked Aβ binding to microglial cells, thus highlighting its neurotherapeutic effect [256]. The protective effects of UA against β-amyloid-induced neurotoxicity were confirmed in pheochromocytoma PC12 cells, where UA protected against Aβ25−35 toxicity by inhibiting caspase-3 activation, reduced cell apoptosis and neuronal cell death [102]. Furthermore, UA administration to Aβ25-35-treated mice improved oxidative stress and inflammatory response [257] by inhibiting NF-κB-dependent inflammation, thus preventing MAPKs phosphorylation [105]. In summary, current evidence suggests that UA possesses potential therapeutic activity in improving insulin sensitivity, hyperglycemia, and inflammation, in addition to its protective role such as receptor modulator, enzyme inhibitor, or in neurotransmitter uptake.

## 3. Conclusions and Perspectives

T2DM and AD share several common abnormalities such as impaired glucose metabolism, increased oxidative stress, insulin resistance, and amyloidogenesis. Accumulation of amyloid-beta peptide observed in AD brains can induce neuronal apoptosis. Similarly, over-expression of amylin (IAPP) promotes amyloid plaque formation that generates death in pancreatic islet β-cells under T2DM stress conditions. The structural and functional integrity of the nervous system can be compromised due to brain insulin resistance and deficiency. AD pathology can enhance brain insulin resistance, and promote neuroinflammation, thus functioning as a positive feedback loop. Numerous studies have demonstrated a positive association between T2DM and AD, although many of the studies were performed using animal and cell models. Based on this positive association, T2DM treatments have been used as a target to diminish or avoid AD onset and progression [258].

Studies addressing the association between AD and T2DM revealed that both diseases are protein misfolding disorders characterized by insulin resistance and abnormal protein aggregation. In addition to its role in metabolic regulation, insulin can inhibit amyloid peptide (Aβ and amylin, IAPP) intracellular accumulation and stimulate degradation by insulin-degrading enzyme (IDE) [259]. In the case of insulin resistance, IDE expression decreases which leads to limited Aβ degradation and thus, indirectly, allowing formation of toxic Aβ aggregates. This imbalance leads to increased levels of amylin in T2DM and Aβ monomers in the AD brain generating toxic oligomers. Besides having an important role in developing function impairments and apoptosis, Aβ peptides compete with insulin on its own receptor, reducing its binding affinity while maintaining an impaired insulin signaling [259].

Insulin receptors are located in both the periphery and central nervous system and have a major contribution in signaling metabolic homeostasis and cell growth. Once insulin binds to its receptors, cJun protein undergoes conformational changes, leading to tyrosine residue phosphorylation. However, this process may be disturbed by pro-inflammatory responses via serine/threonine phosphorylation [260]. Prolonged activation of JNK pathway leading to impaired insulin signaling plays a major role in the development of insulin resistance. Under normal conditions, insulin binds to its receptors, triggers cellular glucose uptake, and reduces Aβ production [261]. However, in insulin-resistant states, perturbed JNK activation has been linked to Aβ peptide accumulation [262] and dysregulation caused by IAPP formation [263]. Based on these mechanisms, pharmacological JNK inhibitors may possess beneficial metabolic effects in preventing inflammation and insulin resistance in both diseases. However, due to low selectivity, JNK drug targets fail to reduce metabolic inflammation and insulin resistance [260]. The proposed used of anti-diabetic, hypoglycemic drugs for the management of AD is driven by the following findings: (i) AD is associated with brain insulin resistance and insulin deficiency; (ii) diabetic patients treated with insulin or hypoglycemic medications exhibit significant improvements in memory by slowing AD progression; (iii) experimental intracerebral or intravenous treatments with insulin improved memory, cognition, evoked brain potentials, and neurotransmitter functions [258]. Although attractive and seemingly simple, insulin-based treatments lower blood sugar and increase risk for problems related to hypoglycemia causing metabolic insults to various organs. Therefore, despite remarkable progress in studying the development of pathology, finding alternative approaches in ameliorating neurodegenerative conditions remains a high priority.

Peptide–drug conjugates are an emerging targeted therapeutics and an exciting area of research that hold great promise [264]. For example, NAP (^1^NAPVSIPQ^8^), a small active fragment of the activity-dependent neuroprotective protein, ADNP (354–361), has been shown to provide neuroprotection at very low concentrations by promoting the dissolution of amyloid plaques and preventing Aβ aggregation, one of the main pathological conditions in AD. Its bioavailability and extended neuroprotection made this peptide a pharmaceutical candidate for both intranasal and intravenous administration routes [265]. Although current literature lacks studies on NAP conjugates, similar compounds that present an acid linked to a peptide revealed pharmacokinetics similar to those reported for drug applications. However, the efficacy and therapeutic applications of small bioactive molecules have been mainly evaluated in preclinical studies, using various cell lines and animal models, thus requiring caution when extending the findings to humans. Considering the limitations of preclinical studies and models including their inability to fully mimic diseases or lack the heterogeneous characteristics of human body, more clinical studies are needed in order to accurately predict the clinical efficacy of the drug candidates as well as to assess their pharmacological safety.

Notwithstanding the above limitations, this review underlines the importance of several acidic compounds in the search for an effective drug against toxicity associated with hyperglycemia and amyloidogenesis. Considering that the purpose of the molecular integration is to achieve superior therapeutic outcomes with diminished adverse effects, it is expected that the novel peptide-conjugates would inherit not only the disaggregation properties of NAP peptide, but will also present characteristics specific to apoptosis inhibitor drugs. Thus, future studies using small compounds, administered as peptide conjugates might offer new treatment options based on combination therapy. The anticipated results could be a macromolecule that will likely inherit both the structure and the properties of its basic components.

## Data Availability

Data sharing not applicable.
